# Alveolar-arterial oxygen gradient: An early marker to predict the severity of community-acquired pneumonia in children

**DOI:** 10.1097/MD.0000000000037670

**Published:** 2024-04-05

**Authors:** Baoxi Zhao, Mingqiang Si, Zhonghua Hu, Junsheng Jiang

**Affiliations:** a Department of Pediatrics, The First People’s Hospital of Linping District, Hangzhou, China.

**Keywords:** alveolar-arterial oxygen gradient, children, community-acquired pneumonia, the Pediatric Respiratory Severity Score

## Abstract

To study the clinical significance of alveolar-arterial oxygen gradient (P_A-a_O_2_) for children with community-acquired pneumonia (CAP). A prospective study was carried out from January 2020 to June 2023, Overall, 100 patients were included in the study, 35 had severe pneumonia, whereas, 65 had non-severe pneumonia. Clinical and laboratory data were retrospectively collected at the time of hospital admission and during hospitalization. Patients were divided into severe and non-severe groups. P_A-a_O_2_ was significantly higher among children with severe pneumonia, as determined by WHO, PRESS (*P* < .001). P_A-a_O_2_ was significantly higher among children with mechanical ventilation, shock, sepsis, and mortality. Receiver operating characteristic curve (ROC) analysis for P_A-a_O_2_ showed that the area under the curve was 0.76 (*P* value < .05), with a sensitivity of 84.3% and a specificity of 67.9%. Our study suggests that P_A-a_O_2_ level has a predictive value for detecting community-acquired pneumonia severity in children.

## 1. Introduction

Community-acquired pneumonia (CAP) in childhood is defined as an acute infection of the pulmonary parenchyma in a child caused by a pathogen acquired outside the hospital, that is, in the community.^[1,2]^ It is an important cause of morbidity in developed countries and an important cause of morbidity and mortality in developing countries.^[3]^ The World Health Organization estimates that approximately 2 million children under the age of 5 years die of pneumonia each year worldwide; the majority of these deaths occur in developing countries.^[4,5]^ The mortality rate in developed countries is less than 1 per 1000 per year.^[1,3]^ Nevertheless, community-acquired pneumonia is associated with enormous costs either directly through medical expenses or indirectly through loss of working hours by parents of sick children.^[6]^

Therefore, one of the main challenges in the management of CAP is to stratify patients early according to their risk of clinical deterioration. alveolar-arterial oxygen gradient (P_A-a_O_2_) is defined as the difference between the alveolar and arteriolar concentration of oxygen, and it is a highly accurate index of pulmonary function.^[7]^ This work attempted to investigate the value of P_A-a_O_2_ in the prediction of CAP severity in pediatric patients.

## 2. Materials and methods

A retrospective study was performed in The First People’s Hospital of Linping District. All CAP patients (35 severe pneumonia, 65 non-severe pneumonia) who were admitted to the pediatric department between January 2020 to June 2023 were enrolled in the study. The study was conducted on children with pneumonia from the age of one month to 14 years. Two patient groups were recruited: the first included children hospitalized with a diagnosis of severe CAP while the second included children with non-severe CAP. All children of their parent gave their consent to participate in the study. The first People’s Hospital of Linping District Ethics Committee approved the study (approval number: linping2023044).

Inclusion criteria were age beyond 1 month up to 14 years and patients diagnosed with CAP, present signs and symptoms of pneumonia. This was confirmed by the radiological finding of a consolidation.^[6]^ Exclusion criteria’s were: severe immune dysfunction; Incomplete clinical data; and Automatic discharge.

We used three severity scores in our research, that is the Respiratory Index of Severity Score (RISC),^[8]^ the Predisposition, Insult, Response, Organ dysfunction modified (PIROm)^[9]^ and the Pediatric Respiratory Severity Score (PRESS).^[10]^

Pediatric Intensive Care Unit (PICU) admission criteria were oxygen saturation (SPO_2_) < 92%, signs of shock, the need for mechanical ventilation, respiratory failure, and altered mental status.^[1]^

The primary outcome measure was the occurrence of the length of PICU stay during the hospital. The secondary outcome measures included shock, mechanical ventilation, and sepsis.

### 2.1. Data collection

Clinical and laboratory parameters of patients were collected from the electronic medical record system. The laboratory findings were analyzed within 3 hours after admission including arterial blood gas analysis and, complete blood count. Furthermore, the severity of pneumonia were determined by mortality predictive scores, including the RISC and the PIROm for respiratory infections, evaluated by the PRESS to determined the severity of respiratory tract infections, clinical criteria and radiological and laboratory data were collected to evaluate score.

### 2.2. Data analyses and statistics

Statistical analyses were performed using SPSS for Windows Version 26 (Chicago, USA). Statistical data are expressed as n (%), and the χ^2^ test. Normally distributed measures are expressed as mean ± standard deviation, and the *t* test for 2 independent samples was used for comparison between groups. The P_A-a_O_2_ was evaluated by the receiver operating characteristic (ROC) curve. The independent risk factors for PICU admission were evaluated. Differences were considered statistically significant at *P* < .05.

## 3. Result

The studied patients consisted of 100 children with CAP, including 65 (65%) with simple pneumonia and 35 (35%) with severe pneumonia. Their main demographic and clinical characteristics are shown in Table 1. Pathogenic bacteria were isolated from blood cultures in only 12 patients (34.3%). Clinical indicators of pneumonia severity, such as the need for mechanical ventilation, shock, and sepsis, presented in the PICU admitted group as 28.6%, 14.3%, and 25.7%, respectively. All patients of the PICU admitted group and 24.6% of patients of the simple pneumonia group had SIRS criteria. These clinical indicators were secondary to CAP.

**Table 1:** Baseline characteristics of the studied population.

	PICU group (n = 35)	Simple pneumonia (n = 65)	*P*
Sex (male, %)	16 (45.7)	36 (55.4)	.381
Age, years	7.42 ± 2.65	7.93 ± 3.67	.687
BMI, kg/m^2^	23.82 ± 2.63	22.28 ± 2.51	.446
Hospital stay (d)	10.25 ± 3.23	6.54 ± 2.58	.001
Mechanical ventilation n (%)	10 (28.6)	0	.001
PICU stay (d)	7.62 ± 3.63	0	.001
shock n (%)	5 (14.3)	0	.001
Sepsis n (%)	9 (25.7)	0	.001
SIRS n (%)	35 (100)	16 (24.6)	.001

Data are presented as mean ± standard deviation, n (%), or median (interquartile range).

BMI = body mass index, PICU = Pediatric Intensive Care Unit.

P_A-a_O_2_ was significantly elevated among PICU admitted compared with simple pneumonia [105.4 (79.4–142.5)] versus [33.8 (23.4–65.8), *P* < .05)]. Acidosis was significantly higher in PICU admitted than in simple pneumonia Table 2.

**Table 2:** Laboratory and radiological characteristics of the studied groups.

	PICU group (n = 35)	Simple pneumonia (n = 65)	*P*
CRP, mg/L	24.45 ± 18.26	23.6 ± 15.28	.28
PCT, ng/mL	2.48 ± 1.33	0.54 ± 0.14	.001
WBC,*10^9^/L	12.28 ± 1.68	11.12 ± 1.26	.36
PH in the arterial blood	7.30 ± 0.13	7.38 ± 0.09	.001
P_A-a_O_2_	105.4 (79.4–142.5)	33.8 (23.4–65.8)	.001

Data are presented as mean ± standard deviation, n (%), or median (interquartile range).

CRP = C-reactive protein, P_A-a_O_2 =_ Alveolar-arterial oxygen gradient, PCT = procalcitonin, WBC = white blood cell.

P_A-a_O_2_ was significantly higher in patients with severe pneumonia complicated with shock, and sepsis and was also higher among those needing mechanical ventilation and who did not survive Table 3.

**Table 3:** P_A-a_O_2_ in relation to patients’ complications and outcomes.

	P_A-a_O_2_, median (IQR)	*P*
Mechanical ventilation		
Yes	92 (64–112)	
No	48 (40–59)	.001
Sepsis		
Yes	80 (58–106)	
No	33 (22–46)	.001
Shock		
Yes	72 (60–84)	
No	42 (33–49)	.001
SIRS		
Yes	76 (64–87)	
No	37 (24–49)	.001
Mortality		
Survivor	115 (96–138)	
Non-survivor	48 (38–64)	.001

Data are presented as median (interquartile range). SIRS systemic inflammatory response syndrome

Pearson correlation analysis showed a positive correlation between P_A-a_O_2_ and RISC score (*r* = 0.764), PRESS score (*r* = 0.786), PIROm score (*r* = 0.725), and duration of hospital stay (*r* = 0.663) (*P* < .01).

**Figure 1.**
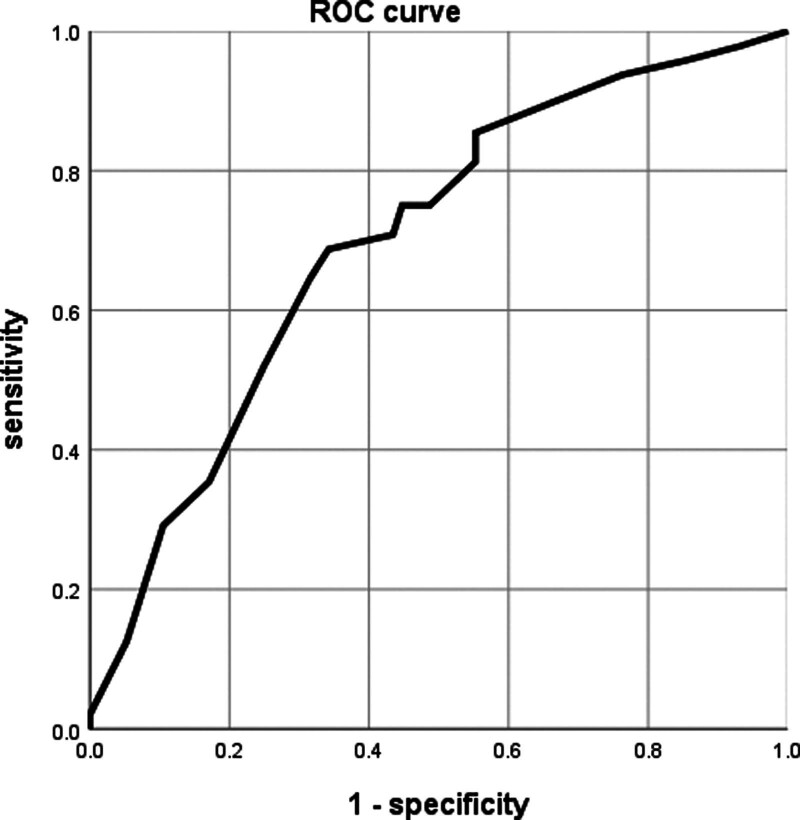
ROC analysis of P_A-a_O_2_ for predicting CAP. CAP = community-acquired pneumonia, P_A-a_O_2_ = Alveolar-arterial oxygen gradient, ROC = receiver operating characteristic curve.

Multivariate regression analysis revealed that P_A-a_O_2_ level, hospital-free days, mechanical ventilation need, shock, and sepsis were independent risk factors for the occurrence of severe pneumonia with odds ratios 6.58, 3.86, 2.85, 2.64, and 1.27, respectively Table 4. The ROC curve analysis for P_A-a_O_2_ level as a predictor of pneumonia severity showed that the area under the curve was 0.76 (*P* value < .05), At a cutoff point (94.6) with a sensitivity of 84.3%, a specificity of 67.9%, positive predictive value of 85.6%, and negative predictive value of 88.9% Figure 1.

**Table 4:** Multivariate regression analysis of risk factors for pneumonia severity.

Factors	OR	95% CI	*P*
P_A-a_O_2_	6.58	3.05–16.38	.001
Hospital-free days	3.86	1.41–6.81	.001
Mechanical ventilation	2.85	1.30–4.74	.001
Shock	2.64	1.64–5.40	.001
Sepsis	1.27	0.88–1.58	.001

CI = confidence interval, OR = odds ratio.

## 4. Discussion

Pneumonia is one of the major causes of hospitalization in children, and CAP plays an important role in the development of pneumonia in children.^[11]^ CAP is mostly self-limiting, but it may also cause life-threatening illness with respiratory distress and multiple organ involvement even in immunocompetent individuals. Severe CAP is characterized by severe respiratory system involvement, multiple system complications, and high mortality.^[12]^ In this retrospective study of 25 subjects diagnosed with severe CAP at our hospital, we preliminarily analyzed the mortality risk factors in children with severe CAP and revealed that P_A-a_O_2_ level, hospital-free days, mechanical ventilation need, shock, and sepsis were independent risk factors for the occurrence of severe pneumonia.

Recently, a study by de Roos et al supported the use of P_A-a_O_2_ in association with chest computed tomography scanning to identify patients in need of hospitalization at an early stage.^[13]^ Moreover, P_A-a_O_2_ at first blood gas analyses at arrival in the hospital was identified as a predictive marker of intensive care unit (ICU) admission.^[14–16]^ The results of the present study align with the data of the consulted literature and suggest that a high P_A-a_O_2_ is a severity marker of severe CAP.

Among children with severe CAP, ventilator-blood flow ratio disorders are present with decreased lung ventilation and ventilation function. Studies have shown that P_A-a_O_2_ can more sensitively reflect lung ventilation function than other commonly used indexes such as oxygen partial pressure and oxygenation index.^[16]^ P_A-a_O_2_ is the difference between alveolar and arterial oxygen partial pressure, and its level is related to ventilation-blood flow ratio, gas dispersion, and anatomic shunt.^[17]^ In the case of severe CAP with stable pulmonary perfusion, the higher the P_A-a_O_2_, the worse the pulmonary gas exchange, and the decreased diffusion function.^[17]^

Clinical indicators of pneumonia severity, such as the need for mechanical ventilation, shock, and sepsis, presented in PICU admitted as 24%, 12%, and 32% respectively. Patients who experienced these clinical indicators were consequent to the development of CAP, which is the most common cause of sepsis in many studies.^[18,19]^ Approximately 40% to 50% of patients with sepsis present respiratory sources of infection.^[20]^In addition, a large adult study suggested that severe sepsis is a common feature in CAP (48% of hospitalized patients), with 4.5% of patients developing septic shock.^[21]^

Regarding P_A-a_O_2_ as a potential prognostic biomarker for pneumonia outcome, we showed that the highest P_A-a_O_2_ level was found among the patients who died. Pipitone G et alnoted that higher circulating levels of P_A-a_O_2_ are associated with higher mortality risk in patients with pneumonia due to the COVID-19 Virus.^[22]^

## 5. Conclusion

In conclusion, P_A-a_O_2_ has a certain sensitivity and specificity for predicting the occurrence of CAP, and have important clinical application value.

## Author contributions

**Conceptualization:** Baoxi Zhao.

**Data curation:** Mingqiang Si.

**Formal analysis:** Mingqiang Si.

**Funding acquisition:** Junsheng Jiang.

**Investigation:** Baoxi Zhao, Mingqiang Si, Zhonghua Hu.

**Methodology:** Baoxi Zhao, Mingqiang Si, Zhonghua Hu.

**Supervision:** Junsheng Jiang.

**Writing – original draft:** Baoxi Zhao, Junsheng Jiang.

**Writing – review & editing:** Baoxi Zhao, Junsheng Jiang.

## References

[1] Bradley JS Byington CL Shah SS.; Pediatric Infectious Diseases Society and the Infectious Diseases Society of America. Executive summary: the management of community-acquired pneumonia in infants and children older than 3 months of age: clinical practice guidelines by the Pediatric Infectious Diseases Society and the Infectious Diseases Society of America. Clin Infect Dis. 2011;53:617–30. 21890766 10.1093/cid/cir625 PMC3202323

[2] Leung AKC Wong AHC Hon KL. Community-acquired pneumonia in children. Recent Pat Inflamm Allergy Drug Discov. 2018;12:136–44. 29932038 10.2174/1872213X12666180621163821

[3] Lanks CW Musani AI Hsia DW. Community-acquired pneumonia and hospital-acquired Pneumonia. Med Clin North Am. 2019;103:487–501. 30955516 10.1016/j.mcna.2018.12.008

[4] Bryce J Boschi-Pinto C Shibuya K.; WHO Child Health Epidemiology Reference Group. WHO estimates of the causes of death in children. Lancet. 2005;365:1147–52. 15794969 10.1016/S0140-6736(05)71877-8

[5] Jain S Williams DJ Arnold SR.; CDC EPIC Study Team. Community-acquired pneumonia requiring hospitalization among U.S. children. N Engl J Med. 2015;372:835–45. 25714161 10.1056/NEJMoa1405870 PMC4697461

[6] Harris M Clark J Coote N.; British Thoracic Society Standards of Care Committee. British Thoracic Society guidelines for the management of community acquired pneumonia in children: update 2011. Thorax. 2011;66(Suppl 2):ii1–23. 21903691 10.1136/thoraxjnl-2011-200598

[7] Avci S Perincek G. The alveolar-arterial gradient, pneumonia severity scores and inflammatory markers to predict 30-day mortality in pneumonia. Am J Emerg Med. 2020;38:1796–801. 32739850 10.1016/j.ajem.2020.05.048

[8] Lee CW Tai YL Huang LM. Efficacy of clarithromycin-naproxen-oseltamivir combination therapy versus oseltamivir alone in hospitalized pediatric influenza patients. J Microbiol Immunol Infect. 2021;54:876–84. 32978076 10.1016/j.jmii.2020.08.017

[9] Reed C Madhi SA Klugman KP. Development of the Respiratory Index of Severity in Children (RISC) score among young children with respiratory infections in South Africa. PLoS One. 2012;7:e27793. 22238570 10.1371/journal.pone.0027793 PMC3251620

[10] Araya S Lovera D Zarate C. Application of a prognostic scale to estimate the mortality of children hospitalized with community-acquired pneumonia. Pediatr Infect Dis J. 2016;35:369–73. 26629871 10.1097/INF.0000000000001018

[11] Julián-Jiménez A Palomo de los Reyes MJ Parejo Miguez R. Improved management of community-acquired pneumonia in the emergency department. Arch Bronconeumol. 2013;49:230–40. 23477946 10.1016/j.arbres.2012.12.008

[12] Yun KW Wallihan R Juergensen A. Community-acquired pneumonia in children: myths and facts. Am J Perinatol. 2019;36(S 02):S54–7. 31238360 10.1055/s-0039-1691801

[13] Chu DK Kim LH Young PJ. Mortality and morbidity in acutely ill adults treated with liberal versus conservative oxygen therapy (IOTA): a systematic review and meta-analysis. Lancet. 2018;391:1693–705. 29726345 10.1016/S0140-6736(18)30479-3

[14] Carlino MV Valenti N Cesaro F. Predictors of Intensive Care Unit admission in patients with coronavirus disease 2019 (COVID-19). Monaldi Arch Chest Dis. 2020;90:458–60. 10.4081/monaldi.2020.1410 32672430

[15] de Roos MP Kilsdonk ID Hekking PW. Chest computed tomography and alveolar-arterial oxygen gradient as rapid tools to diagnose and triage mildly symptomatic COVID-19 pneumonia patients. ERJ Open Res. 2021;7:00737–2020. 33718488 10.1183/23120541.00737-2020 PMC7898029

[16] Moammar MQ Azam HM Blamoun AI. Alveolar-arterial oxygen gradient, pneumonia severity index and outcomes in patients hospitalized with community acquired pneumonia. Clin Exp Pharmacol Physiol. 2008;35:1032–7. 18518885 10.1111/j.1440-1681.2008.04971.x

[17] Harris DE Massie M. Role of Alveolar-Arterial Gradient in Partial Pressure of Oxygen and PaO_2_/Fraction of Inspired Oxygen Ratio Measurements in Assessment of Pulmonary Dysfunction. AANA J. 2019;87:214–21. 31584399

[18] Angus DC Linde-Zwirble WT Lidicker J. Epidemiology of severe sepsis in the United States: analysis of incidence, outcome, and associated costs of care. Crit Care Med. 2001;29:1303–10. 11445675 10.1097/00003246-200107000-00002

[19] Alberti C Brun-Buisson C Chevret S.; European Sepsis Study Group. Systemic inflammatory response and progression to severe sepsis in critically ill infected patients. Am J Respir Crit Care Med. 2005;171:461–8. 15531752 10.1164/rccm.200403-324OC

[20] Beutz MA Abraham E. Community-acquired pneumonia and sepsis. Clin Chest Med. 2005;26:19–28. 15802162 10.1016/j.ccm.2004.10.015

[21] Dremsizov T Clermont G Kellum JA. Severe sepsis in community-acquired pneumonia: when does it happen, and do systemic inflammatory response syndrome criteria help predict course? Chest. 2006;129:968–78. 16608946 10.1378/chest.129.4.968

[22] Pipitone G Camici M Granata G. Alveolar-arterial gradient is an early marker to predict severe pneumonia in COVID-19 Patients. Infect Dis Rep. 2022;14:470–8. 35735760 10.3390/idr14030050 PMC9222321

